# Mitigation of detraining effects: physical activity improves protein profile and physical function of aged amid COVID-19

**DOI:** 10.1186/s12877-025-06347-0

**Published:** 2025-09-29

**Authors:** Renato Jimenez Gomez, Fernanda Rodrigues Monteiro, Angélica Rodrigues Botelho, Ariane Nardy, Fernanda Cristina da Silva, Millena Soares de Almeida, Vitória Paixão, Brenda Rodrigues Silva, Marcelo Rossi, Jônatas Bussador do Amaral, Gislene Rocha Amirato, Carlos André Freitas Santos, Guilherme Eustáquio Furtado, Rodolfo de Paula Vieira, Ana Paula Ribeiro, Luiz Henrique da Silva Nali, André Luis Lacerda Bachi

**Affiliations:** 1 Post-Graduation Program in Health Sciences, Santo Amaro University, Rua Isabel Schmidt349 - Santo Amaro, São Paulo, 04743-030 SP Brazil; 2 Faculty of Pharmacy, Santo Amaro University, Campus, São Paulo, Brazil; 3 Ear, Nose and Throat (ENT) Lab, Department of Otorhinolaryngology, Federal University of São Paulo (UNIFESP), São Paulo, Brazil; 4 Mane Garrincha Sport Education Center, Sports Department of the Municipality of São Paulo (SEME), São Paulo, Brazil; 5 Discipline of Geriatrics and Gerontology, Department of Medicine, Paulista School of Medicine, Federal University of Sao Paulo (UNIFESP), São Paulo, Brazil; 6 Polytechnic Institute of Coimbra, Applied Research Institute, Rua da Misericórdia, Lagar dos Cortiços-S, Martinho do Bispo, Coimbra, Portugal; 7 SPRINT - Sport Physical activity and health Research & INnovation cenTer, Polytechnic Institute of Coimbra, Coimbra, Portugal; 8 Research Centre for Natural Resources Environment and Society (CERNAS), Polytechnic Institute of Coimbra, Bencanta, Coimbra, Portugal; 9 Human Movement and Rehabilitation Post Graduation Program, Evangelical University of Goiás (UniEVANGELICA), GO, Brazil

**Keywords:** SARS-CoV-2, Social isolation, Albumin, health aging, physical exercise

## Abstract

**Aim:**

We investigated the impact of abrupt interruption, imposed by COVID-19, and subsequent resumption of regular practice of a combined exercise training program on protein, renal profile, and physical functional capacity in physically active older adults before the pandemic.

**Methods:**

Thirty-five volunteers (mean age 64.36 ± 19.43 years) participated, with data collected before the COVID-19 pandemic (PRE), 12 months after social isolation (PAN, detraining period), and 10 months after returning to regular exercise (POST).

**Results:**

In the POST period, both older women (*n* = 26) and men (*n* = 9) exhibited higher serum levels of albumin and total protein (*p* < 0.0001 for albumin and total proteins in older women group; *p* = 0.0033 for albumin and *p* = 0.0002 for total proteins in older men group) compared to PRE and PAN. Older women showed, both at PAN and POST, elevated serum creatinine levels (*p* = 0.0309; *p* = 0.0003, respectively) and Timed Up and Go Test (TUGT) values (*p* = 0.0096; *p* = 0.0013, respectively), along with decreased estimated creatinine clearance in PAN (*p* = 0.0132; *p* = 0.0005, respectively) compared to PRE. Additionally, older women demonstrated lower gait speed (*p* = 0.0210) and muscle strength (*p* = 0.0002) in POST compared to PAN and PRE. Older men exhibited higher serum creatinine levels and estimated creatinine clearance in POST compared to PRE. Significant correlations between biochemical parameters, estimated creatinine clearance, and physical functional tests were observed, particularly in older women.

**Conclusions:**

Overall, the study suggests that resuming combined exercise training partially mitigated the detraining-induced impairment in metabolic and physical capacities among older adults.

**Supplementary Information:**

The online version contains supplementary material available at 10.1186/s12877-025-06347-0.

## Introduction

Aging is a natural, multifaceted process that affects individuals differently, resulting in increased rates of morbidity and mortality among older adults [1]. Biological and gender factors contribute to this difference, with women generally living longer than men. However, women may experience greater frailty despite showing lower biological age in some biomarkers. Conversely, men often demonstrate better physical-functional fitness status [2]. It is crucial to recognize that certain habits and lifestyles, including sedentary behavior, malnutrition, and declining physical function, play central roles in influencing the aging process and are associated with development and progression of several chronic conditions, such as sarcopenia and frailty [3, 4].

The coronavirus disease 19 (COVID-19) pandemic, originated by the novel coronavirus SARS-CoV-2, until now, has affected global public health [5]. Following the World Health Organization’s (WHO) declaration of the COVID-19 pandemic [6], widespread implementation of quarantine measures and social distancing protocols aimed at curbing the virus has led to significant alterations in daily life, such as the interruptions to regular practice of physical activity and exercise regimens [7]. Thus, the pandemic-related restrictions, such as gym closures (indoor) and limitations on outdoor activities, have led to a sharp decline in physical exercise practice by the overall population, mainly older adults [8]. Regarding aged people, who are vulnerable to age-related health issues, not only the pandemic-induced restrictions have posed additional challenges, exacerbating physical, immunometabolism, and mental health concerns, but also the abrupt interruption in regular exercise routines imposed deleterious effects on systemic health related to nutritional status, including disruptions to protein profiles, renal function, and physical functional capacities [8–10].

Low body mass index (BMI) and serum albumin levels are pillars of poor nutritional status and have been linked to an increased risk of disability and physical-functional deterioration in old age [11, 12]. Likewise, assessment of circulating levels of total protein, urea, and creatinine provides valuable insights into metabolic disturbances and musculoskeletal status, including the presence of muscular disorders associated with protein catabolism [13]. Additionally, evaluating renal function through systemic creatinine levels and creatinine clearance is essential, especially in older adults, given the prevalence of malnutrition and metabolic disturbances, which are core elements of frailty in this population [14].

Among strategies for avoiding and managing chronic conditions include regular physical exercise [15, 16], since it contributes to the improvement and maintenance of body systems, particularly the musculoskeletal and cardiovascular systems, thereby promoting overall health. Physical exercise is widely recognized as a cornerstone of healthy aging, playing crucial roles in maintaining physical function, metabolic health, and mental well-being including older adults [17].

Although it has been reported the impact of the interruption of exercise training practice on some metabolic parameters and physical functional capacities in older adults [9], there remains a lack of information on the specific effects of resuming physical exercise practice on these variables in aged people. Therefore, we investigated the effects of both the interruption and the subsequent return to regular practice of combined physical exercise program during the COVID-19 pandemic on the systemic protein and renal profile, as well as in physical function tests in physically active older adults before the pandemic, in order to improve our understanding about the effects of the COVID-19 lockdown in this population, after all, other lockdowns such it may be implemented in the future.

## Materials and methods

### Study design

This retrospective, longitudinal, observational, descriptive, open study with blind analysis of outcomes followed the Strengthening the Reporting of Observational Studies in Epidemiology (STROBE) guidelines to ensure methodological rigor and transparent reporting [18]. It was assessed: anthropometric data, systemic metabolic parameters, physical function tests, and muscle strength in a group of older adults.

### Participants recruitment and settings

The study involved community-dwelling older adults who were physically active before the COVID-19 pandemic. In this respect, it is worth mentioning that the volunteers regularly practiced the combined exercise training program for at least 10 years (with a maximum of 20 years) at the same local, and they resumed practicing the exercise training as soon as the social isolation measures were eased. The participants were recruited from the Primary Health Care Program for Older Adults at the Universidade Federal de São Paulo (UNIFESP) and practiced a combined physical exercise program at the “Mané Garrincha” Sports and Education Center in São Paulo, Brazil.

### Selection criteria of participants

Volunteers eligible for participation in the study met specific inclusion criteria, which included (i) regular involvement in the combined exercise training program, with a minimum adherence of 75% in the exercise sessions per semester, an official rule of the local in which the exercise training was performed; (ii) being over 60 years old; (iii) autonomy in commuting to the exercise program’s center; (iv) and medical clearance for participation. Conversely, exclusion criteria encompassed individuals with chronic infections, neoplasia, liver, neurological, or kidney diseases, as well as those with type 1 diabetes mellitus, recent use of anti-inflammatory drugs or corticosteroid therapy, or infection by the SARS-CoV-2 virus during the study period. These criteria were essential to ensure the homogeneity of the study population and minimize confounding factors that could affect the outcomes of interest.

### Sample size calculation

Based on the values of TUGT reported in our previous study [9], the Cohen’s *d* effect size was calculated, resulting in an effect size of d = 0.96. Converting this value to an effect size *f* for use in the one-way ANOVA for repeated measures, the calculated effect size was *f* = 0.59. Taking into account this effect size (0.59), the G*Power analysis was carried out considering a power of 0.95 [19], an alpha level of 0.05, a correlation among repeated measures of 0.3, and a nonsphericity correction of 0.5, thus resulting in a minimum sample size of 20 participants. Considering a 30% margin for potential dropouts, the required sample size increases to 26 participants. It is worth mentioning that, although this sample size was reached for the older women group (*n* = 26), the number of volunteers in the older men group (*n* = 9) did not reach this sample size, indicating that this sample was underpowered as a standalone analysis. However, we opted to retain this volunteer group analysis in the study in order to provide complementary insights alongside the older women group, recognizing that these findings should be interpreted with caution.

### Participants enrollment

**Fig. 1:**
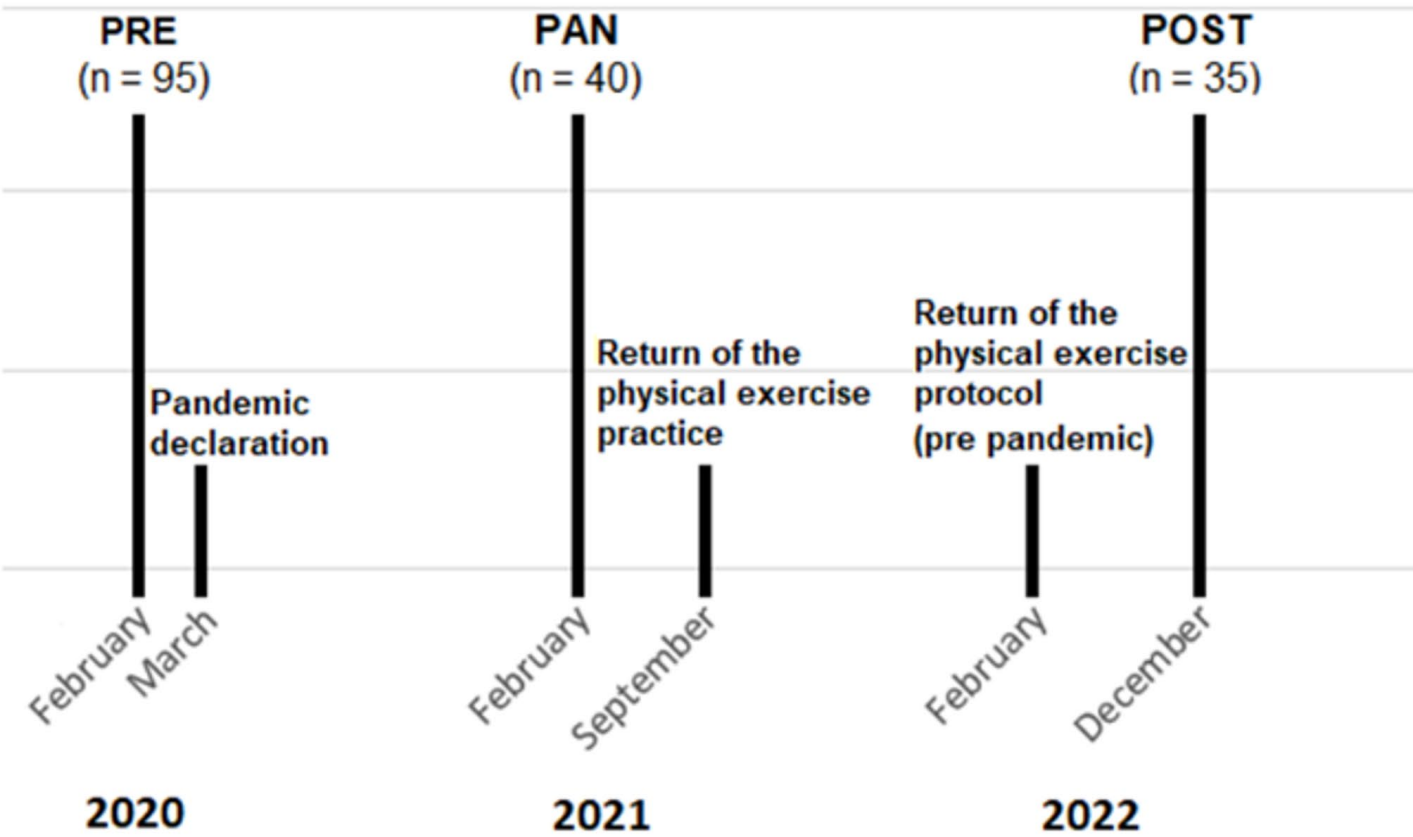
Study design, mainly highlighting the time points in which the blood samples and volunteers data were collected (PRE, PAN, and POST) in accordance with the COVID-19 pandemic

As shown in Fig. 1, during the first week of February 2020, before WHO declared the COVID-19 pandemic, clinical and physical data, as well as biological samples, from a cohort of 95 older adults were obtained in order to develop a previous study. However, one month after, when the WHO officially declared the COVID-19 pandemic, this previous study was interrupted. Subsequently, our research group proposed the current study, and one year later (in February 2021), we extended invitations to the same volunteers, resulting in the participation of only 40 individuals, due to changes of address, and/or fear of being contaminated by SARS-CoV-2 during the evaluations. As social isolation restrictions gradually eased and older adults were allowed to resume regular exercise training in 2022, we contacted the same individuals again, and only a total of 35 volunteers, being 26 older women and 9 older men, reported the resumption of pre-pandemic exercise routines. As previously mentioned, although only the older women group reached the sample size required, we also retained the older men group in the study. Thus, the study’s outcomes are based on data and samples collected from these 35 older adults, which occurred: before the pandemic (PRE), 12 months after the social isolation (PAN, detraining period), and 10 months following the resumption of regular exercise training (POST).

### Ethical procedures

Volunteers provided informed consent approved by the Research Ethics Committees of UNIFESP and at the Universidade Santo Amaro (UNISA) (approval number 3.623.247 and approval numbers 4.350.476 and 6.231.052, respectively), in accordance with the Declaration of Helsinki and ethical guidelines outlined in Resolutions 466/2012 and 510/2016 of the Brazilian National Health Council [20, 21].

### Outcomes measures

#### Anthropometric assessments

Anthropometric data collection procedures adhered to standardized and validated techniques as previously described [22]. This included accurate measurement of body weight using a digital scale (Personal^®^ scale, Filizzola, São Paulo, Brazil) with an accuracy of 0.1 kg, and body height using a wall stadiometer with precision to the nearest 0.1 cm. BMI, calculated as weight divided by height squared [BMI = weight/height^2], was derived from these measurements. Chronological age was regarded as a continuous variable in the study.

#### Physical function assessments

Physical function assessments included the Timed-up-go test (TUGT, in seconds) and gait speed (meters/second) [23–25], conducted following established protocols and guidelines from the European Working Group on Sarcopenia in Older People (EWGSOP) [26]. Muscle strength was evaluated through the handgrip (HG) test (kilograms of force) using an analog dynamometer (Jamar Hydraulic Hand Dynamometer^®^), with the best performance recorded from three attempts with a 1-minute interval between each, always performed by the participant’s dominant hand [26].

#### Blood sampling collection

As previously mentioned, peripheral blood samples were collected in tubes containing anticoagulant EDTA on PRE, PAN and POST. Plasma aliquots were obtained after centrifuging the blood tube (300 g, 10 min at 4 °C) and were used to evaluate systemic levels of total protein, albumin, urea, and creatinine. It is noteworthy that volunteers were instructed to complete their last exercise training session 24 h before blood sampling collections.

#### Laboratory assessments

Circulating concentrations of total proteins and albumin were determined using commercial kits (Bioclin-Quibasa, Belo Horizonte, Minas Gerais, Brazil), with the results analyzed by an automated system (Dimension^®^ RxL Max^®^ Integrated Chemistry System, Siemens, Deerfield, IL, USA). In addition, the circulating concentrations of urea and creatinine were determined on the semi-automated analyzer COBAS INTEGRA 400 plus, by using reagents from the manufacturer Roche^®^ (Roche Diagnostics GmbH, brand Cobas). Lastly, the estimated creatinine clearance, also called Creatinine Clearance, was determined using the Cockcroft-Gault equation [27].

#### Physical exercise intervention

The combined exercise training (CET) program was designed, conducted, and developed by skilled professionals, based on American College of Sport Medicine (ACSM) guidelines [28], which can be found in our previous reports [29, 30]. Briefly, the CET consists of a blend of sets, repetitions and rest intervals of muscle strength-resistance combined to aerobic exercises, conducted for 60–75 min per session at moderate intensity, three times a week on alternate days. Consistent supervision was provided by the same physical education professional throughout both the pre-pandemic and post-isolation exercise sessions.

#### Statistical treatment

The normality of data distribution was initially assessed using the Shapiro-Wilk test, followed by an examination of variance homogeneity using Levene’s test. Parametric variables, presented as mean and standard deviation, were analyzed using one-way ANOVA for repeated measures with the Student-Newman-Keuls post-hoc test. Non-parametric variables, presented as median and interquartile ranges (25–75%), were analyzed using the Friedman test with the Müller-Dunn post-hoc test. Both Pearson’s and Spearman’s rank correlation coefficients were applied to verify the associations between all parameters evaluated in this study. The significance level was set at 5% (*p* < 0.05) and all statistical analyses were performed using the GraphPad version 8.0 program.

## Results

### Demographic and anthropometric data

Table 1 presents demographic and anthropometric data across three assessed occasions. Only age showed a statistically significant difference in both volunteer groups over the years.

**Table 1:** Demographic (age) and anthropometric data (weight, height, and body mass index – BMI) of the older adult population participating in the study on three different occasions: before the COVID-19 (PRE); 12 months after social isolation imposed by the COVID-19 pandemic (PAN); and 10 months after the return of regular practice of combined exercise training (POST)

Women (*n* = 26)				*p* value	Men (*n* = 9)				*p* value
Variables	PRE	PAN	POST		PRE		PAN	POST	
**Age (years)**	73.5 ± 5.68*^,#^	74.5 ± 5.68^§^	75.3 ± 5.71	< 0.0001	72.0 ± 6.24*^,#^		73.0 ± 6.14^§^	74.6 ± 6.24	< 0.0001
**Weight (kg)**	62.20 ± 12,7	62.15 ± 13.5	62.15 ± 12.7	NS	70.10 ± 8.42		67.81 ± 9.78	70.18 ± 8.16	NS
**Height (m)**	1.55 ± 0.06	1.53 ± 0.07	1.53 ± 0.07	NS	1.68 ± 0.05		1.69 ± 0.07	1.69 ± 0.07	NS
**BMI (kg/m** ^**2**^ **)**	26.29 ± 4.28	26.20 ± 4.52	25.93 ± 4.33	NS	24.81 ± 3.07		23.88 ± 2.46	24.83 ± 2.84	NS

Note: NS = non-significant. * = significant differences in relation to the values found in the PAN time point. # = significant differences in relation to the values found in the POST time point. § = significant differences in relation to the values found in the POST time point

### Proteic and kidney profile

**Fig. 2:**
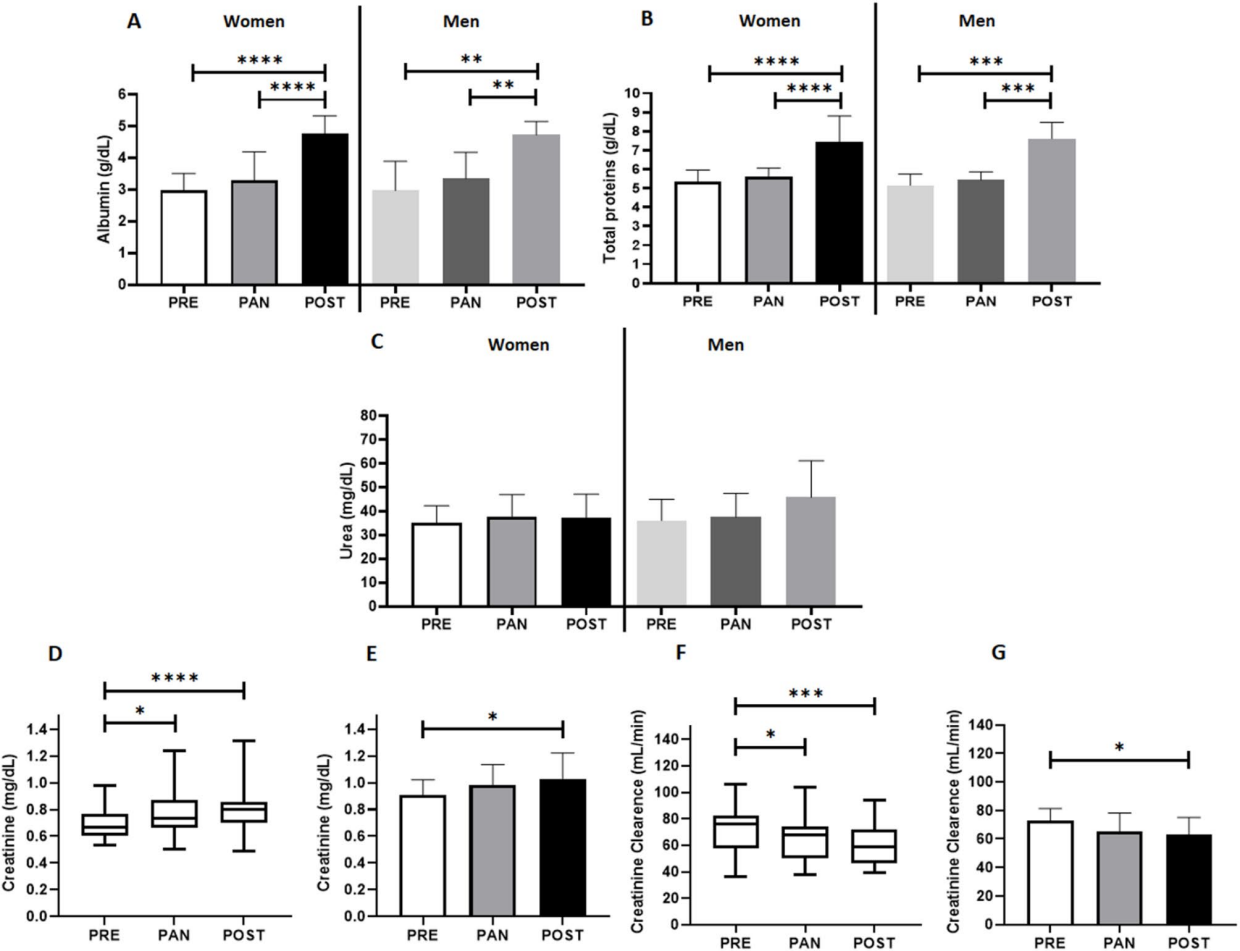
Serum concentration of albumin (g/dL, **A**), total proteins (g/dL, **B**), urea (mg/dL, **C**), creatinine (mg/dL, older women group - **D** and older men group - **E**), and values of estimated creatinine clearance (mL/min, older women group - **F** and older men group - **G**) at the time points assessed in the study (pre-pandemic = PRE, 12 months after social isolation imposed by COVID-19 pandemic = PAN; and 10 months after the return of the regular practice of combined exercise training = POST). Note: *p = < 0.05; **p = < 0.01; ***p = < 0.001; ****p = < 0.0001

Figure 2 illustrates the circulating concentrations of albumin (Fig. 2A), total proteins (Fig. 2B), urea (Fig. 2C), creatinine (Fig. 2D), and creatinine clearance (Fig. 2E) in volunteer groups. Higher systemic concentrations of albumin and total proteins were observed at POST occasion than the other two occasions in both groups (*p* < 0.0001 for albumin and total proteins in older women, and *p* = 0.0033 for albumin and *p* = 0.0002 for total proteins in older men). Older women exhibited increased circulating creatinine concentrations in PAN (*p* = 0.0309) and POST (*p* = 0.0003) compared to PRE, while older men showed an elevation only at POST (*p* = 0.0455). Moreover, estimated creatinine clearance decreased significantly in older women during PAN (*p* = 0.0132) and POST (*p* = 0.0005), and in older men only at POST (*p* = 0.0245), with no differences in urea concentrations in either group.

### Physical functional tests

**Fig. 3:**
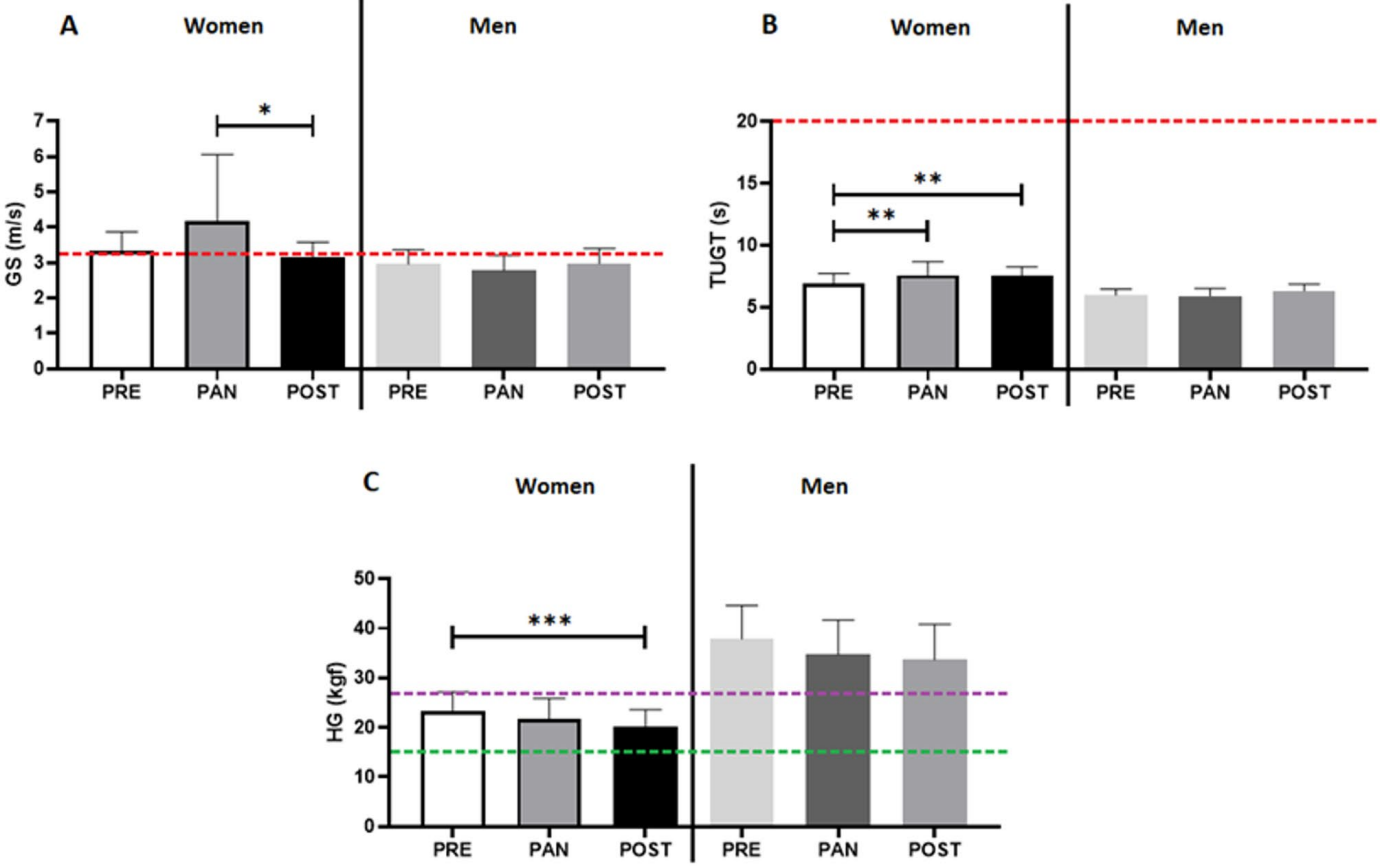
Values of the physical function tests, Gait Speed (GS in meter per seconds - m/s, **A**), Time Up and Go Test (TUGT in seconds, **B**), muscle strength (Handgrip - HG, in kilograms force - kgf, **C**) at the time points assessed in the study (pre-pandemic = PRE, 12 months after social isolation imposed by COVID-19 pandemic = PAN; and 10 months after the return of the regular practice of combined exercise training = POST). Notes: *p = < 0.05; **p = < 0.01; ***p = < 0.001. The red dashed line represents the score limit for the sarcopenia diagnosis in older populations according to the EWGSOP, i.e. >3.2 m/s for GS and 20 s for TUGT. The green dashed line represents the score limit for the sarcopenia diagnosis in the HG test for women (< 16 kgf), and the purple dashed line represents the score limit for the sarcopenia diagnosis in the HG test for men (< 27 kgf), both according to the EWGSOP.

Figure 3 shows the data concerning the physical functional tests GS (Figure 3A), TUGT (Figure 3B) and muscle strength [handgrip (HG), Figure 3C)]. The older women group presented higher GS values during PAN than POST (p=0.0210). Moreover, TUGT values were elevated in PAN (p=0.0096) and POST (p=0.0013) as compared to PRE. Conversely, no differences were observed in these parameters for older men. In the HG test, the older women group exhibited lower values in POST than PRE (p=0.0002), whereas the older men group showed no significant difference.

### Correlation analysis

**Fig. 4:**
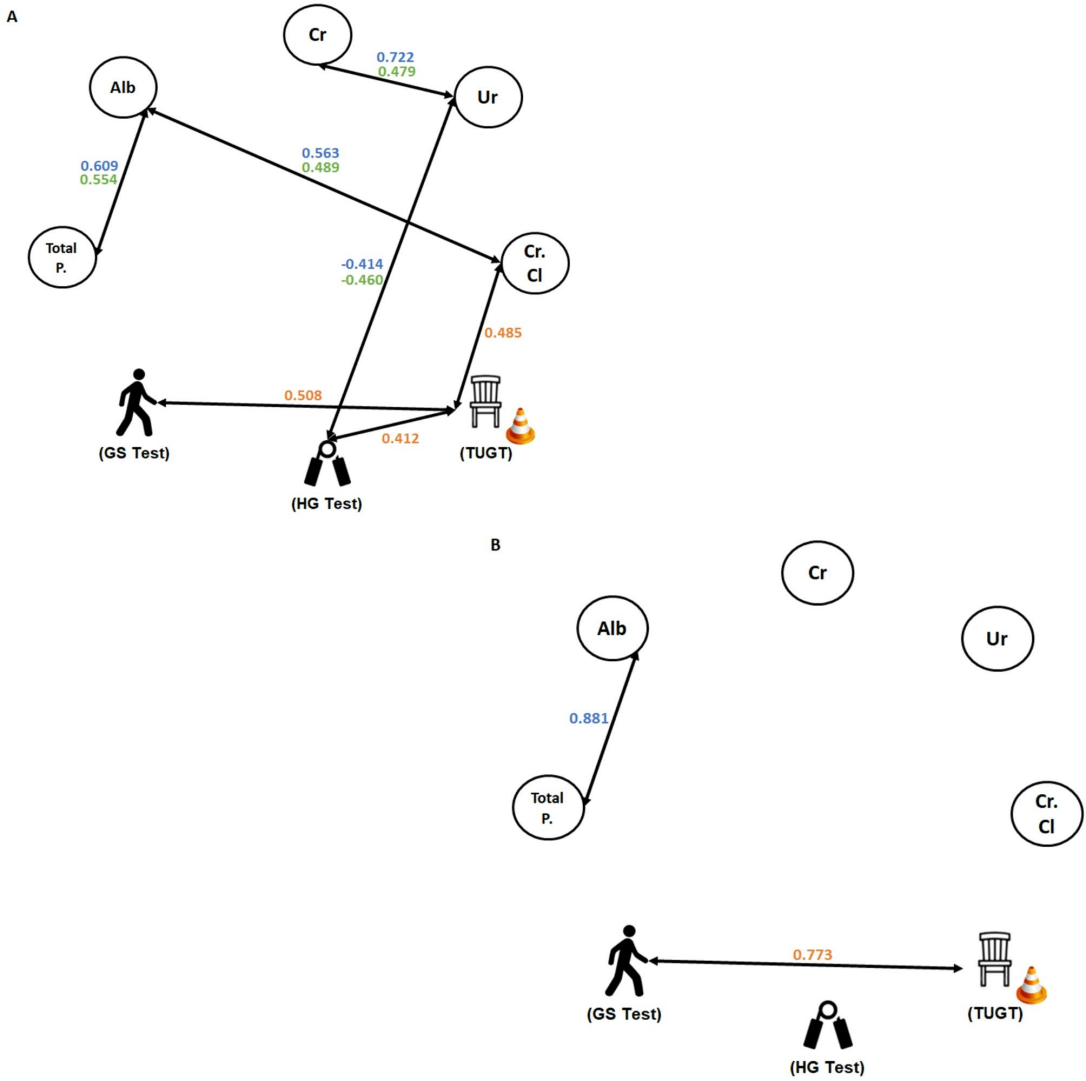
Spearman coefficient correlation analysis between the parameters assessed here in the older women group (**A**) and older men group (**B**). Notes: The green color represents the “rho” values found in the PRE time point. The orange color represents the “rho” values found in the PAN time point. The blue color represents the “rho” values found in the POST time point. Alb = Albumin; Total P. = Total Proteins; Cr = Creatinine; Ur = urea; Cr. Cl = creatinine clearance; GS = Gait Speed; HG = Handgrip; TUGT = Time Up and Go Test

Figure 4 displays correlation analysis results for both volunteer groups. Regarding older women group (Fig. 4A), significant positive correlations were observed between creatinine and urea in the PRE (*p* = 0.013) and POST (*p* < 0.0001) occasions; albumin and total proteins or creatinine clearance in the PRE (*p* = 0.003 and *p* = 0.011, respectively) and POST (*p* = 0.001 and *p* = 0.019, respectively) occasions; TUGT and GS (*p* = 0.011), or HG (*p* = 0.045), or creatinine clearance (*p* = 0.016) in the PAN occasion. Significant negative correlations were found between HG test values and urea concentrations in the PRE (*p* = 0.021) and POST (*p* = 0.040) occasions. Besides, Fig. 4B displays positive correlations in older men between TUGT and GS test (*p* = 0.015), as well as total proteins and albumin (*p* = 0.002), in PAN and POST occasions, respectively.

## Discussion

In general, our results highlight two key outcomes: firstly, the interruption of physical exercise during the COVID-19 pandemic led to notable alterations in some parameters associated with systemic protein profile and physical functional capacity, particularly in the older women group; secondly, upon the resumption of physical exercise practice 10 months later, circulating concentrations of albumin and total protein, as well as in the GS test were improved. However, both volunteer groups also exhibited a decline in muscle strength test performance and creatinine clearance.

Despite the most significant findings were obtained in the older women group, it is with mentioning that they not only could have been influenced by the number of female volunteers, but also may be associated with the disparities across biological, developmental, social, and behavioral aspects, which lead to notable implications on outcomes such as the development and progression of chronic diseases, comorbidities, and mortality rates [31, 32]. Corroborating our findings, it was shown that women exhibit greater impairment in physical and functional aspects than men, contributing to higher rates of conditions such as sarcopenia and frailty among women [33, 34].

In line with findings from previous findings [35, 36], our results showed that a 12-week interruption of physical exercise practice led to detraining effects here, mainly evidenced by muscle strength and functional mobility reductions, which were more pronounced in older women. Additionally, a significant positive correlation between GS and TUGT was verified in both volunteer groups during this occasion, suggesting exacerbation of detraining effects on physical function in older adults. Contrary to previous observations [37], a positive correlation between TUGT and HG test in older women was found. While further research is needed, these findings suggest differential effects of detraining on upper and lower limb muscle strength in older adults.

It is of utmost importance to note that, although some studies reported that adults had exercised more, especially performing outdoor walking/running and cycling, during the COVID-19 lockdown [38, 39], people aged 55 + and older self-reported to exercise less during the lockdown, which impacted not only in their level of activity but also in their general and mental health [39, 40]. These pieces of information are aligned with the self-reports provided by our volunteers, since during the COVID-19 isolation period, whenever possible, they practiced walking as the main option to maintain their physically active lifestyle. However, walking was not practiced regularly, as it was discouraged both by public health policies and by their families. Additionally, in their own homes, the limitation of resources, in conjunction with little motivation, led them to find it difficult to practice any physical activity.

Interestingly, the progressive decline in HG values, observed in our study, provides insights into the variations observed in these physical function tests, mainly during the POST period, when the volunteers returned to maintain a consistent, regular practice of CET. This suggests that the 10-month interval was insufficient to reverse or mitigate the observed decline. Even though the physical exercise program included resistance training, it failed to induce the muscle plasticity necessary for performance recovery after detraining. Other factors, such as epigenetic, metabolic, and nutritional influences, can have also hindered neuromuscular adaptation and performance improvement in our study population [41].

Although there is a lack of data concerning nutritional aspects, the circulating protein biomarkers assessment provides valuable insights into metabolic and musculoskeletal status, aiding in the identification of metabolic and muscular disorders, particularly those associated with protein catabolism. Serum albumin levels, for instance, can serve as sensitive indicators of health in the aged population, since albumin lower levels are associated with indicators of malnutrition, increased morbidity and mortality, as well as are correlated with higher systemic concentrations of pro-inflammatory cytokines in older adults [42, 43].

Here, we found a simultaneous increase in systemic serum albumin and total protein levels during the POST period than both the PRE and PAN occasions, which reinforces that serum albumin is the predominant protein in plasma [44]. Additionally, significant positive correlations between serum albumin and total protein levels were observed in both volunteer groups during the PRE and POST occasions. These findings suggest a potentially pivotal role of CET in regulating these circulating protein biomarkers in the study’s participants.

However, the discrepancy observed in serum levels of total protein and, particularly, albumin between the PRE and POST occasions can suggest two potential explanations. Firstly, the lower circulating albumin levels in the PRE occasion may indicate its role in maintaining systemic inflammatory responses at a healthy level. Secondly, the elevated serum levels of these biomarkers in the POST occasion could be attributed to improved protein intake during the PAN and POST periods [45, 46].

Besides assessing systemic levels of albumin and total protein, we also evaluated serum levels of urea and creatinine [43], which are indicative of musculoskeletal status and play roles in renal function [47, 48]. Regarding serum urea levels, our findings revealed that their values remained within the normal range (10–45 mg/dL) throughout the study, which can suggest the absence of musculoskeletal and renal disturbances among participants [49, 50].

Notably, negative significant correlations between circulating urea levels and handgrip (HG) test values were observed in both the PRE and POST occasions, particularly in the older women group. This suggests that higher muscle strength is associated with lower serum urea levels and indicates the potential regulatory role of physical exercise in this association.

As formerly mentioned, creatinine serves as a valuable biomarker for renal function and skeletal muscle mass in healthy populations [27, 51, 52]. Here, we observed a gradual increase in serum creatinine levels and a corresponding decrease in estimated creatinine clearance during the PAN and POST occasions, especially in the older women group, although these alterations remained within the normal range for healthy aged individuals. Creatinine, a byproduct of creatine metabolism in muscles, is eliminated via free filtration in the kidneys [53]. Of note, serum creatinine levels may overestimate skeletal muscle mass and underestimate renal function decline due to physiological reductions in glomerular filtration rate, particularly with aging. Furthermore, the estimated creatinine clearance calculated through the Cockcroft-Gault equation offers greater reliability in assessing renal function [27, 54] aligning with our findings and the expected decline in glomerular filtration rate over time, especially among women.

Despite our results are consistent with existing literature, factors such as protein intake, exercise duration, and hydration status can influence creatinine bioavailability and estimated renal clearance calculation [27, 55]. Moreover, the unexpected positive correlation between estimated creatinine clearance and TUGT values during the PAN period in the older women group contrasts with previous findings and suggests a potential impairment due to detraining in those population [56, 57]. These findings highlight the multifaceted nature of creatinine dynamics and the importance of considering various factors influencing its interpretation, particularly in the context of aging and exercise interventions.

Regarding BMI, it serves as a widely accepted indirect marker of nutritional status in older adults [45] and, here, their values remained unchanged throughout the study. In fact, BMI values falling between 20 and 22 kg/m² are indicative of potential risks of fractures, functional dependence, hospitalization, and increased mortality in this population [45]. According to the WHO classification [58], whereas the older women group presented as overweight (25.0–29.9 kg/m²), the older men group presented normal (adequate) weight (18.5–24.9 kg/m²). However, specifically for the older adult population, the Brazilian Food and Nutritional Surveillance System (SISVAN) and the Brazilian Health Ministry suggests a different BMI classification, where values ranging from 22 to 27 kg/m² are considered indicative of adequate weight [59, 60]. Based on it, all participants in our study fell within this range, underscoring their overall healthy nutritional status.

It is worth citing that, the possibility of evaluating the effect of detraining, imposed by the COVID-19 lockdown, on a robust older adult population, followed by the resumption of regular CET practice, not only was useful to provide valuable insights on older adults’ physical functional capacity and metabolic parameters, but also could be use to support preventive and intervention actions in order to motivate and encourage the older adults in maintaining the exercise practice during a lockdown, since it is known that physical inactivity negatively affects the health of this population, even though it is necessary to mention that this was an observational study and the findings should be interpreted with caution.

In sum, the strengths of the study was include comprehensive assessments of circulating biomarkers related to protein profile and physical function tests, providing a multifaceted view of participants’ health status, especially by the inclusion of older women and men, which improves the study’s generalizability, as well as the use of validated techniques for data collection and statistical analysis that enhances the reliability and validity of the findings. Besides, it is paramount to cite that the study presents some limitations, such as (a) the lack of evaluation of certain metabolites in urine samples to assess renal function, which could have provided a more comprehensive understanding of participants’ health; (b) the inability to compare data with other populations, such as sedentary older adults or younger individuals; (c) the absence of information regarding dietary habits and systemic inflammatory status of volunteers; (d) the lower sample size verified in the older men group, although it imposes on us to interpret their results with caution, the maintenance of this group was useful for providing complementary insights alongside the older women group; and (e) the absence of a better measurement concerning the physical activity performed by the volunteers during the pandemic period, since some of them only self-reported that, during the quarantine, they were performing walking, which may also have influenced some of the outcomes observed in the study.

## Conclusion

Our study presents novel findings indicating that 10 months of regular physical exercise practice, initiated following a year of COVID-19-induced social isolation (detraining period), resulted in improvements in GS values and circulating levels of protein metabolites, particularly albumin and total protein, among physically active older women and men. However, despite these positive outcomes, other physical functional parameters affected during the detraining period were not fully restored by the resumption of physical exercise. These findings underscore the importance of regular physical exercise practice in mitigating the adverse effects of detraining, especially in older adults, while also highlighting the need for continued research to understand the long-term impacts of physical exercise interruption on health and functional outcomes in this population.

## Supplementary Information

Below is the link to the electronic supplementary material.

Supplementary Material 1

## Data Availability

The raw data supporting the conclusions of this article will be made available by the authors, without undue reservation, under requirement at the following email: jgrenato@hotmail.com.
