# Supplementary material for: Mitigation of detraining effects: physical activity improves protein profile and physical function of aged amid COVID-19
Source: BMC Geriatr. 2025 Sep 29;25:738. doi: 10.1186/s12877-025-06347-0 (PMC12482374; doi:10.1186/s12877-025-06347-0)
Supplement: Supplementary file 1 — Supplementary Material 1 [file 12877_2025_6347_MOESM1_ESM.pdf]

TERMO DE CONSENTIMENTO LIVRE E ESCLARECIDO

Convidamos o(a) Sr(a) para participar do projeto de pesquisa intitulado "**IMPACTO DA SOROPOSITIVIDADE PARA CITOMEGALOVÍRUS (CMV) E DO PERFIL DA MICROBIOTA NA RESPOSTA IMUNE/INFLAMATÓRIA SISTÊMICA E NAS VIAS AÉREAS SUPERIORES DE IDOSOS VACINADOS PARA DOENÇAS RESPIRATÓRIAS**", sob a responsabilidade do pesquisador e docente do Programa de Pós-Graduação em Ciências da Saúde da Universidade de Santo Amaro - UNISA, o Prof. Dr. André Luis Lacerda Bachi e com colaboração da Disciplina de Geriatria e Gerontologia e do Centro de Estudos do Envelhecimento, ambos pertencentes à Universidade Federal de São Paulo (UNIFESP).

Fica esclarecido que sua participação se dará por meio de adesão voluntária ao processo de seleção e recrutamento realizado pelo médico geriatra colaborador do estudo.

Vale informar que atualmente o mundo está diante de uma pandemia originada pelo novo coronavírus SARS-CoV-2, causador da doença denominada como "doença de coronavírus-2019" ou COVID-19. Mais recentemente, em nosso país, um surto de gripe, originada pelos vírus H1N1 e H3N2, tem aumentado o número de casos de internação ou mesmo a procura por cuidados médicos e assistenciais. Dentre vários aspectos, tanto para COVID-19 quanto para gripe, é sabido que a população idosa se apresenta não apenas como a população mais afetada, mas também que esta contabiliza o maior número de mortes por estas doenças. Estudos sugerem que o aumento da taxa de infecção, gravidade e letalidade pelos vírus SARS-CoV-2 e *Influenza* em idosos está associada tanto à ocorrência da imunossenescência quanto do fenômeno "inflammaging". Neste sentido, tem sido proposto que uma microbiota com predominância de agentes patogênicos bem como a reativação da infecção pelo citomegalovírus (CMV), um herpes vírus, podem favorecer o desenvolvimento da imunossenescência, pelo fato deste poder ser um importante fator desencadeante do "inflammaging". Vale destacar que é sabido que a imunossenescência e o "inflammaging" impactam de forma negativa nas respostas vacinais dos idosos. Diante dessas informações, este estudo objetiva investigar o impacto da soropositividade para CMV e do perfil da microbiota sobre as respostas imunes/inflamatórias sistêmicas e de vias aéreas superiores em idosos vacinados para a COVID-19 e gripe.

Para podermos responder ao objetivo deste estudo, duzentos (n=200) idosos, de ambos os sexos, com idade entre 60 a 85 anos, serão convidadas a participar do estudo. O recrutamento, seleção e obtenção dos dados antropométricos, físicos e clínicos dos voluntários do estudo ocorrerão junto ao Ambulatório de Promoção de Saúde do Idoso da UNIFESP, entidade co-participante do estudo. Já, sobreo plano de trabalho do presente estudo, vale esclarecer que para o desenvolvimento do estudo serão utilizadas amostras de: 1) saliva, que será coletada através do uso de tubos salivette; 2) sangue, que será obtido pela

punção de sangue venoso periférico em tubos secos e com anticoagulante ETDA; e 3) fezes. As amostras serão coletadas em dois momentos: antes e 30 dias depois da vacinação para a COVID-19 e gripe, sendo estas utilizadas para: avaliação de características imunológicas, inflamatórias, e da microbiota residente na cavidade oral e intestinal. Importante informar também que todas as amostras biológicas coletadas serão devidamente processadas e armazenadas nas dependências do Centro de Pesquisas da UNISA ou UNIFESP até que sejam utilizadas nas avaliações laboratoriais preconizadas no estudo. Vale destacar que as amostras inicialmente não utilizadas nas avaliações laboratoriais permanecerão armazenadas no mesmo local para repetição das análises, caso seja necessária comprovação dos resultados, ou mesmo para que outras análises possam ser realizadas, sendo neste caso solicitado sua permissão.

Os riscos decorrentes de sua participação na pesquisa são mínimos, podendo haver pequeno desconforto no momento da coleta das amostras de material biológico, como, por exemplo, formação de pequeno hematoma (mancha roxa) no momento de coleta de sangue. Além disso, a manifestação de constrangimento ao se expor durante a realização da coleta de material biológico ou mesmo alterações na autoestima provocadas pela evocação de memórias sobre sua condição clínica podem configurar-se como menor risco. Será garantido sigilo a respeito dos nomes e dos resultados individuais de cada participante, sendo os dados obtidos neste estudo agrupados para confecção dos relatórios, trabalhos de conclusão de curso, dissertações e artigos científicos. Todos os voluntários terão acompanhamento clínico pelos médicos geriatras colaboradores do estudo, com total acesso aos respectivos resultados.

Se o(a) Sr(a) aceitar participar, estará contribuindo não só compreender melhor como a infecção pelo CMV e alterações na microbiota podem impactar nas alterações nas respostas imunes, inflamatórias no idoso vacinado contra a COVID-19 e gripe, mas também poderá permitir a implantação de novas estratégias de promoção de saúde para esta população.

É garantido o acesso, em qualquer etapa do estudo, aos profissionais responsáveis pela pesquisa para esclarecimento de eventuais dúvidas ou informações sobre os resultados parciais das pesquisas, quando em estudos abertos, ou de resultados que sejam do conhecimento dos pesquisadores.

Se você tiver alguma consideração ou dúvida sobre a ética da pesquisa, o Sr(a) pode entrar em contato com o Comitê de Ética em Pesquisa da UNISA (CEP-UNISA) localizado na Rua Prof. Enéas de Siqueira Neto, 340, Jardim das Imbuías,SP - Telefone (11) 2141-8687 de Segunda a Sexta, das 08hs às 13hs.

É garantida sua a Vossa Senhoria a liberdade da retirada de seu consentimento a qualquer momento e assim deixar de participar do estudo, sem qualquer prejuízo à continuidade de qualquer benefício que você tenha obtido junto às Instituições, antes, durante ou após o período deste estudo. As informações obtidas pelos pesquisadores serão analisadas em conjunto com as de outros participantes, não sendo divulgada a identificação de nenhum deles. Não há despesas pessoais para o participante em qualquer fase do estudo, incluindo exames e consultas. Também não há compensação financeira relacionada à sua participação. Se existir qualquer despesa adicional, ela será absorvida pelo orçamento da pesquisa. (No caso de

ressarcimento de despesas dos participantes da pesquisa e delas decorrentes, tais como transporte e alimentação, explicitar como ocorrerá esse ressarcimento e suas condições – Res. 466/12.II.21).

Em caso de dano pessoal, diretamente relacionado aos procedimentos deste estudo (anexo causal comprovado), a qualquer tempo, fica assegurado ao participante o respeito a seus direitos legais, bem como procurar obter indenizações por danos eventuais.

Uma via deste Termo de Consentimento ficará em seu poder.

São Paulo,_____/____/_____ ____________________________________

Prof. Dr. André Luis Lacerda Bachi

(Pesquisador Principal)

Se você concordar em participar desta pesquisa assine no espaço determinado abaixo e coloque seu nome e o nº de seu documento de identificação.

Nome: (do participante): ....

Doc. Identificação: ....

Ass: ....
